# Editorial of the Special Issue “Toxins: Mr Hyde or Dr Jekyll?”

**DOI:** 10.3390/toxins15020142

**Published:** 2023-02-10

**Authors:** Daniel Ladant, Gilles Prévost, Michel R. Popoff, Evelyne Benoit

**Affiliations:** 1 Institut Pasteur, Unité Biochimie des Interactions Macromoléculaires, 25-28 Rue du Docteur Roux, F-75015 Paris, France; 2 Institut de Bactériologie, Unité UR-7290 Virulence Bactérienne Précoce, ITI InnoVec, 3 Rue Koeberlé, F-67000 Strasbourg, France; 3 Institut Pasteur, Unité Toxines Bactériennes, 25-28 Rue du Docteur Roux, F-75015 Paris, France; 4 CEA, Institut des Sciences du Vivant Frédéric Joliot, Département Médicaments et Technologies pour la Santé (DMTS), Service d’Ingénierie Moléculaire pour la Santé (SIMoS), Université Paris-Saclay, EMR 9004 CNRS/CEA, F-91191 Gif-sur-Yvette, France

The 27th Annual Meeting of the French Society of Toxinology (SFET, http://sfet.asso.fr/international (accessed since 1 September 2022)) was held on 9–10 December 2021 as a virtual meeting (e-RT27). The central theme selected for this meeting, “Toxins: Mr Hyde or Dr Jekyll?”, gave rise to three thematic sessions: the first on plant toxins, algal toxins and mycotoxins; the second on animal toxins; and the third on bacterial toxins. All sessions were aimed at emphasizing the latest findings on this topic. Apart from this central theme, a “miscellaneous” session was dedicated to recent results obtained in toxinology. Ten speakers from seven countries (Australia, Brazil, Burkina Faso, France, Germany, The Netherlands and the United States of America) were invited as international experts to present their work, and other researchers and students presented theirs through 15 shorter lectures and 20 posters. Of the ca. 80 participants who registered, 38% were foreigners, a value highlighting the international attractiveness of the SFET meeting.

The Special Issue “Toxins: Mr Hyde or Dr Jekyll?” includes ten articles.

All the abstracts of the ten invited speaker’s lectures, as well as those of the 12 shorter lectures and 20 posters, are available in the report from the 27th Meeting of Toxinology [1]. Owing to a donation from MDPI‘s *Toxins*, two prizes of 300EUR each were awarded to the best oral communication and best poster, both selected from a vote by all the invited speakers to the meeting and the SFET Board of Directors. The two winners of the best oral communication award (Barbara Ribeiro: Functional impact of BeKm-1, a high-affinity hERG blocker, on cardiomyocytes derived from human-induced pluripotent stem cells) and from the best poster award (Anne-Cécile Van Baelen: Characterization of immune animal toxins for the functional study of angiotensin receptors) are mentioned in the meeting report [1].

Clostridial neurotoxins, botulinum neurotoxins (BoNTs) and tetanus neurotoxins (TeNTs) are produced by sporulating and anaerobic bacteria from the environment. The synthesis of clostridial neurotoxins is a highly regulated process by complex regulatory networks including alternative sigma factors, two-component systems, non-coding RNA, quorum-sensing system, and the interplay with general metabolism. Environmental and nutritional factors control the regulation of clostridial neurotoxin synthesis but are still poorly understood. Notably, amino acid-to-peptide metabolism transition seems to be an important regulatory factor. Comparative regulation of BoNT and TeNT synthesis is reviewed in the article of Popoff and Brüggemann [2].

Various bacterial pathogens produce toxins that target cyclic nucleotide monophosphate (CNMPs). Certain toxins exhibit nucleotidyl cyclase activities that are activated by eukaryotic factors (adenylate cyclase (AC) of *Bordetella pertussis* CyaA, or *Bacillus anthracis* edema factor EF). Davi et al. propose a robust and efficient in vitro assay for the detection of AC activity based on the spectrometry detection of cyclic AMP after chromatographic separation on aluminum oxide. This assay is very sensitive (fmol level) and can be used in complex media [3].

Bacterial toxins and virulence factors are involved in potent pathogenicity mechanisms. One of the host targets is the immune system. However, hosts have generated sensors to detect the pathogenic potential of microbes. Indeed, the innate immunity senses microbial-associated molecular patterns (MAMPs), notably virulence factors, to distinguish between pathogens and commensals. The innate immunity can persist in host and it is called the innate immune memory of trained immunity. The toxins and virulence factors that target Rho-GTPases activate the NLRP3 inflammasome. The release of interleukins, such as IL-1β and IL-18, are associated with the induction of the adaptive immune memory. The innate immunity and trained immunity triggered by virulence factors that modify Rho-GTPases are discussed in the article by Torre and Boyer [4].

A particular group of bacterial toxins concern the toxin superantigens. These toxins recognize specific receptors on both antigen-presenting cells and T lymphocytes leading to the activation of a large proportion of T lymphocytes (up to 20%). Toxin superantigens are involved in several mild-to-severe diseases in humans and animals. Toxin superantigens are mainly produced by *Staphylococcus aureus*. They constitute a very heterogeneous family ranging from 20 to 89% in similarity. The review by Truant et al. concerns the repertoire of *Staphylococcus* superantigens including their functional and pathological properties [5].

*Clostridium botulinum* C and D produce a toxin or enzyme, called C3, that specifically inactivates the eukaryotic protein Rho by ADP-ribosylation. C3 enters only certain cell types such as monocyte-derived cells including macrophages, osteoclasts, and dendritic cells. Thus, inactivated C3 at the enzymatic site can be used as a transporter for the specific delivery of cargo into macrophages and dendritic cells. Fellermann et al. investigated the selectivity of C3 delivery by using eGFP coupled to non-toxic C3. Thereby, C3 is a promising molecule to specifically deliver small compounds into macrophages and dendritic cells [6].

The killer strains of *Debaryomyces hansenii* and *Wickerhamomyces anomalus* species secrete antimicrobial proteins called killer toxins active against selected fungal phytopathogens. Czarnecka et al. investigated the role of plasma membrane pleiotropic drug-resistant (PDR) transporters (Pdr5p and Snq2p) in the mechanism of defense against killer toxins. They showed that the Snq2p efflux pump influences the immunity to the *W. anomalus* BS91 killer toxin and that the activity of the killer toxins of *D. hansenii* AII4b, KI2a, MI1a and CBS767 strains is regulated by other transporters than those influencing the *W. anomalus* killer toxin activity. This is the first study that reports the involvement of PDR transporters in the cell membrane of susceptible microorganisms in resistance to killer yeasts’ toxins [7].

Gambierol is a polycyclic ether toxin produced by the dinoflagellate *Gambierdiscus toxicus* that is a potent neurotoxin by blocking voltage-gated potassium channels. Benoit et al. have analyzed the effect of gambierol on single rat fetal (F19–F20) adrenomedullary-cultured chromaffin cells [8]. Using whole-cell voltage-clamp recording, gambierol was shown to block only a fraction of the total outward K^+^ current by slowing down their activation kinetics. It was found that gambierol did not affect calcium-activated K^+^ (K_Ca_) or ATP-sensitive K^+^ (K_ATP_) channels. These results highlight the important modulatory role played by K_Ca_ channels in the control of exocytosis from fetal (F19–F20) adrenomedullary chromaffin cells.

Acid-sensing ion channels (ASICs) are voltage-independent H^+^-gated cation channels largely expressed in the nervous system of rodents and humans. At least six isoforms (ASIC1a, 1b, 2a, 2b, 3 and 4) associate into homo- or heterotrimers to form functional channels with highly pH-dependent gating properties. Verkets et al. reviewed the pharmacological profiles of animal peptide toxins targeting ASICs, including PcTx1 from tarantula and related spider toxins, APETx2 and APETx-like peptides from sea anemone, and mambalgin from snake, as well as the dimeric protein snake toxin MitTx that have all been instrumental to understanding the structure and the pH-dependent gating of rodent- and human-cloned ASICs and to study the physiological and pathological roles of native ASICs in vitro and in vivo [9]. ASICs are expressed all along the pain pathways and the pharmacological data clearly support a role for these channels in pain.

α-Bungarotoxin is a large, 74 amino acid toxin containing five disulfide bridges, initially identified in the venom of *Bungarus multicinctus* snake. Similar to most large toxins, the chemical synthesis of α-bungarotoxin is challenging, explaining why all previous reports use purified or recombinant α-bungarotoxin. However, only chemical synthesis allows easy insertion of non-natural amino acids or new chemical functionalities. Herein, Brun et al. described a procedure for the chemical synthesis of a fluorescent-tagged α-bungarotoxin [10]. An azide-modified Cy5 fluorophore was coupled to the toxin. Using automated patch-clamp recordings, the fluorescent synthetic α-bungarotoxin blocked acetylcholine-mediated currents in response to muscle nicotinic receptor activation in TE671 cells. Thus, synthetic fluorescent-tagged α-bungarotoxin retains excellent properties for binding onto muscle nicotinic receptors.
